# From Agro-Industrial Waste to Food Safety: Sustainable Biochars Derived from Orange Peel and Guava Leaves for the Removal of Aflatoxin B1 in Poultry Feed Using an In Vitro Model

**DOI:** 10.3390/foods15173005

**Published:** 2026-08-26

**Authors:** Karla S. García-Salazar, Raquel López-Arellano, Juan D. Latorre, Elvia Adriana Morales Hipólito, Jorge L. Mejía-Méndez, Edgar R. López-Mena, Alma Victoria Sánchez-Mendoza, Alma Vázquez-Durán, Guillermo Tellez-Isaias, Abraham Méndez-Albores, Bruno Solis-Cruz, Daniel Hernandez-Patlan

**Affiliations:** 1 Nanotechnology Engineering Division, Polytechnic University of the Valley of Mexico, Tultitlan 54910, State of Mexico, Mexico; karla.garcia.salazar@upvm.edu.mx (K.S.G.-S.); alma.sanchez@upvm.edu.mx (A.V.S.-M.); 2 Laboratory 5: LEDEFAR, Multidisciplinary Research Unit, Superior Studies Faculty at Cuautitlan (FESC), National Autonomous University of Mexico (UNAM), Cuautitlan Izcalli 54714, State of Mexico, Mexico; lopezar@unam.mx (R.L.-A.); eadriana_mh@comunidad.unam.mx (E.A.M.H.); 3 Division of Agriculture, Department of Poultry Science, University of Arkansas, Fayetteville, AR 72701, USA; jl115@uark.edu; 4 Escuela de Ingeniería y Ciencias, Tecnologico de Monterrey, Epigmenio González 500, San Pablo, Santiago de Querétaro 76130, Querétaro, Mexico; mejia.jorge@tec.mx; 5 Escuela de Ingeniería y Ciencias, Tecnologico de Monterrey, Av. General Ramón Corona 2514, Zapopan 45138, Jalisco, Mexico; edgarl@tec.mx; 6 Unidad de Investigación Multidisciplinaria L14-A1 (Ciencia y Tecnología de Materiales), Facultad de Estudios Superiores Cuautitlán (FESC), National Autonomous University of Mexico (UNAM), Cuautitlan Izcalli 54714, State of Mexico, Mexico; almavazquez@comunidad.unam.mx (A.V.-D.); albores@unam.mx (A.M.-A.); 7 Gut Healt LLC, Fayetteville, AR 72703, USA; gtellez@uark.edu

**Keywords:** agro-industrial waste, orange peel, guava leaves, biochar, aflatoxin B1, removal efficiency, poultry feed, in vitro models

## Abstract

Aflatoxin B1 (AFB1) contamination of feed used in poultry farming is an important issue in food safety since it compromises animal productivity and leads to the transfer of toxic waste to the food chain. In this sense, as an alternative to conventional mineral adsorbents, in the present study, two sustainable and low-cost biochars were obtained from agro-industrial waste of orange peel (B-OP) and guava leaves (B-GL) to evaluate their efficiency in the removal of AFB1 in an in vitro avian model. Biochars were obtained from pyrolysis and characterized in terms of particle size, surface area, morphology, surface charge, surface chemistry, and pore size. Furthermore, their adsorption capacity was evaluated in an avian in vitro model. The results showed that B-OP had a smaller particle size (55.30 µm), a larger specific surface area (31.50 m^2^/g), and a smaller pore size (2.38 nm) than B-GL (82.88 µm, 9.74 m^2^/g, and 6.24 nm). Furthermore, the biochars presented different morphologies, FTIR spectra, and zeta potentials. In the avian in vitro model, the feed matrix reduced the effectiveness of AFB1 removal compared to the in vitro model using only buffer solutions. However, B-OP (27.4%) significantly outperformed B-GL (22.7%) in the intestinal segment. The valorization of these agro-industrial wastes into biochar represents an economical and sustainable strategy for removing AFB1 and strengthening food security.

## 1. Introduction

The poultry industry has established itself as one of the fastest growing livestock sectors worldwide in recent decades, being a fundamental pillar in satisfying the growing demand for high-quality animal protein (meat and eggs) in the face of nutritional demands driven by global demographic growth and the income levels of the population [1,2,3]. However, in modern poultry production systems, high productive performance must be achieved, as well as ensuring the quality of products and by-products, animal health, and food safety throughout the production chain [4]. In this sense, preventing contamination of poultry feed with biological and/or chemical agents has become a fundamental priority in the poultry industry, as it has direct effects on productive efficiency, the quality of products and by-products, animal welfare, and even human health [5].

Among feed contaminants, mycotoxin-producing fungi are some of the most important, since these secondary metabolites are toxic to animals and humans even at low concentrations [6]. Although various mycotoxins can be produced in poultry feed, the occurrence levels of aflatoxins are the highest (64–100%) compared to those of other mycotoxins [7]. Aflatoxins are classified into B1, B2, G1, and G2 and are highly toxic metabolites produced by various species of filamentous fungi of the genus Aspergillus, specifically A. flavus and A. parasiticus [8]. However, aflatoxin B1 (AFB1) is the most predominant and important in terms of toxicity in broiler chickens because it causes oxidative damage, intestinal barrier dysfunction, dysbiosis, impaired nutrient adsorption, immunosuppression, hepatotoxicity, and enzyme dysfunction in target organs, which negatively affects the production parameters of broiler chickens (lower weight gain and feed efficiency), reduces egg production and hatchability, and increases mortality and susceptibility to infectious diseases [9,10,11].

In addition to reducing poultry productivity, AFB1 poses a significant food safety challenge because residues can accumulate in tissues and eggs after prolonged exposure [12]. Therefore, controlling AFB1 contamination in feed is crucial to reducing human exposure to carcinogenic aflatoxins through the food chain. In this context, different approaches have been proposed for its control, which involve physical, chemical, and biological methods such as irradiation, ozonation, ammoniation, enzymatic degradation, microbial detoxification, and chemical inactivation [13]. However, the remarkable thermal and chemical stability of AFB1 makes its complete removal through conventional feed processing technologies difficult [14]. Furthermore, several of these methods can reduce the nutritional quality of the feed or generate undesirable degradation products [14]. Consequently, the incorporation of adsorbent materials into feed has become the most practical, economical, and widely implemented strategy to reduce the gastrointestinal bioavailability of AFB1 in poultry production systems [15].

Although adsorbent materials such as clays, zeolites, and aluminosilicates have been used for years as a strategy to control AFB1, there are limitations to their complete use since they tend to be less specific and their effects can be reversed [15]. Furthermore, these materials can bind to important nutrients, thus decreasing the nutritional content of feed, and contain highly toxic impurities that can affect animal health [16]. Accordingly, the use of biochars derived from agro-industrial waste has emerged as a promising alternative to address the problems arising from AFB1 in poultry production, as they exhibit excellent adsorbent properties due to their high specific surface area, well-developed porous structure, abundant oxygen-containing functional groups, and excellent physicochemical stability [17,18]. In fact, biochars have been reported to be as efficient as bentonite clays, or even more efficient than them, at adsorbing mycotoxins, including AFB1 [19]. Furthermore, biochars have been reported to have the ability to adsorb a broader spectrum of mycotoxins than clays, which have a high affinity for AFB1 but exhibit limited binding to other mycotoxins [20]. Among the different agro-industrial wastes used to obtain biochars due to their high contents of hemicellulose, cellulose, and lignin (85–90%) [21], orange peels generated by the citrus industry and guava leaves produced during orchard pruning and fruit harvesting represent abundant lignocellulosic wastes, which can be used for applications related to the removal of contaminants [22,23].

The citrus industry generates millions of tons of waste annually from citrus processing, representing between 50% and 60% of the total mass of citrus fruits, making it one of the largest sources of agro-industrial waste [24,25]. Recently, guava, a fruit widely distributed in tropical and subtropical regions (Brazil, Mexico, China, India, and North Africa), has gained increasing attention due to its rich nutritional composition [26]. Consequently, the demand for guava production has led to an increase in annual leaf biomass production, resulting in agro-industrial waste that can be repurposed [27]. In this sense, there is a need to revalue these agro-industrial wastes to effectively and economically convert them into high-value products with industrial and commercial potential, thus reducing their environmental impact [24,27].

Therefore, the objective of the present study was to obtain two sustainable and low-cost biochars from agro-industrial waste of orange peel (B-OP) and guava leaves (B-GL) to evaluate their efficiency in the removal of AFB1 in an in vitro avian model. Unlike conventional adsorption studies conducted solely in buffer solutions and under temperature and contact time conditions that do not represent physiological conditions [28], this model simulates the dynamic gastrointestinal conditions of broilers, including feed presence, pHs, enzyme activity, and residence time in the crop, proventriculus, and an intestinal section [29]. Furthermore, this model provides a more realistic assessment of adsorbent performance and achieves greater success in animal studies.

## 2. Materials and Methods

### 2.1. Chemicals and Reagents

Aflatoxin B1 (AFB1, purity ≥ 98%) was purchased from Cayman Chemical Company (Ann Arbor, MI, USA), whereas dimethyl sulfoxide (DMSO, purity ≥ 99.5%, analytical grade) was purchased from Sigma-Aldrich (Saint Louis, MO, USA). Acetonitrile (ACN, HPLC grade, purity ≥ 99.9%), methanol (MeOH, HPLC grade, purity ≥ 99.9%), hydrochloric acid (HCl, reagent grade, purity 36.5–38.0%), sodium hydroxide (NaOH, reagent grade, purity ≥ 98.0%), and sodium bicarbonate (NaHCO_3_, reagent grade, purity 99.7–100.3%) were purchased from JT Baker (Radnor, PA, USA). Pepsin (1:10,000) and pancreatin (8×) were obtained from Bio Basic (Markham, ON, Canada). Water was purified using a Milli-Q system (Merck-Millipore, Darmstadt, Germany).

### 2.2. Preparation of Biochars

The agro-industrial waste used to obtain the biochars consisted of orange peel derived (B-OP) from oranges collected in Tihuatlán, Veracruz, Mexico, and guava leaves (B-GL), which were collected in Tultitlán, State of Mexico, Mexico. Both agro-industrial residues were carefully washed with distilled water to remove surface impurities and organic matter and were left to dry at room temperature for two weeks to remove moisture. These agro-industrial residues were subsequently subjected to a controlled carbonization process in a muffle furnace (Marla J-15, MARLA Equipos, Nezahualcóyotl, Mexico) at 360 °C for 60 min in the presence of oxygen to remove volatile compounds and promote the formation of initial carbonaceous structures with an incipient pore network.

After carbonization, the resulting material was removed from the muffle furnace and cooled to room temperature. The carbonized material was then ground in a mortar to reduce the particle size and obtain a fine powder. The activation stage involved subjecting the material to a second heating at 720 °C for 60 min to develop a porous structure and generate active adsorption sites. Finally, after cooling to room temperature, samples were sieved through a No. 35 mesh (500 μm) to standardize the particle size.

### 2.3. Characterization of Biochars

#### 2.3.1. Particle Size Determination by Laser Diffraction

The particle size of B-OP and B-GL was determined using a laser diffraction analyzer equipped with a Tornado dry powder system (LS 13 320; Beckman Coulter, Miami, FL, USA). This particle size analyzer featured a 5 mW laser diode with a wavelength of 750 nm. The samples were loaded into a plastic cylinder to achieve an obscuration value between 4% and 8%. Data were collected and analyzed using LS 13 320 XR ADAPT software version 1.2.323 (Beckman Coulter, FL, USA).

#### 2.3.2. Scanning Electron Microscopy (SEM)

Morphological analysis of B-OP and B-GL was performed using a JEOL JSM-6010LA scanning electron microscope (JEOL Ltd., Tokyo, Japan). Microscopic analysis was performed at 100×, 500×, and 1000× magnification with an accelerating voltage of 10.0 kV and a working distance of 14 mm. Before analysis, each sample was mounted on a copper slide with a carbon tape.

#### 2.3.3. Fourier Transform Infrared Spectroscopy with Attenuated Total Reflection (FTIR-ATR)

FTIR spectra of B-OP and B-GL were obtained using a Frontier SP8000 FTIR spectrophotometer (Perkin Elmer, Waltham, MA, USA) equipped with an ATR accessory (DuraSamplIR II, Smiths Detection, Warrington, UK) over a scan range of 450–4000 cm^−1^, with an average of 32 scans and a resolution of 4 cm^−1^. A background spectrum was collected before the BCS, and baseline and ATR corrections were applied to the spectrum for further analysis.

#### 2.3.4. Point of Zero Charge (pH_pzc_)

The zero-charge point was determined following a previously described methodology with slight modifications [30]. Briefly, 10 mg of each biocarbon (B-OP and B-GL) was placed in three tubes, and 10 mL of deionized water adjusted to different pH values (1, 3, 5, 7, 9, and 11, initial pH) was added. The tubes were shaken at 200 rpm for 195 min at room temperature to reach equilibrium, and the final pH of the supernatants was measured using a Mettler Toledo SevenMulti potentiometer (Mettler Toledo, Schwerzenbach, Switzerland) equipped with a combined glass electrode. The pH_pzc_ was calculated by plotting the difference in pH (ΔpH: pH_final_ − pH_initial_) against the initial pH, and the point at which the line crosses the *x*-axis represents the pH_pzc_ of the biochar.

#### 2.3.5. Zeta Potential

The electrokinetic potential of B-OP and B-GL was determined by laser Doppler velocimetry (LDV), that is, by electrophoretic mobility, using a Zetasizer instrument (ZetaSizer Pro, Malvern Instruments, Worcestershire, UK), following the recommendations of Ramales-Valderrama et al. [31]. The determinations were performed in triplicate at different pH values (1, 3, 5, 7, 9, and 11) at a temperature of 25 °C with an equilibration period of 120 s, and each measurement comprised 11 cycles to obtain a stable reading. The results were analyzed using ZS Xplorer software (Malvern Panalytical Ltd., Malvern, UK).

#### 2.3.6. Determination of Surface Area and Pore Size

Nitrogen adsorption/desorption isotherms were obtained at −195.8 °C using a NOVA 4200e surface area and pore size analyzer (Quantachrome Instruments, Boynton Beach, FL, USA). Before analysis, the samples were degassed at 350 °C for 12 h to remove moisture and other adsorbed gases from the surface. The specific surface area of the sample was calculated using the Brunauer–Emmett–Teller (BET) method, and the pore size distributions were calculated using the BJH (Barrett–Joyner–Halenda) method from the desorption branch of the isotherms.

### 2.4. Determination of AFB1 by Ultra-High-Performance Liquid Chromatography (UPLC)

AFB1 quantification was performed using an Acquity H-Class UPLC^®^ system equipped with a quaternary pump system, an autosampler, and a photodiode array detector (PDA; Waters, Milford, MA, USA). For chromatographic analysis, an Acquity UPLC^®^ HSS T3 column (100 mm × 2.1 mm, 1.8 μm; Waters Corporation, Milford, MA, USA) was used at a temperature of 40 °C. The mobile phase consisted of a mixture of water, MeOH, and ACN (55:25:20) at an isocratic flow of 0.3 mL/min. The sample injection volume was 20 μL, with a total analysis time of 5 min. Detection was performed at a wavelength of 360 nm, and data acquisition and processing were performed using Empower 3 software (Waters, 2010, Milford, MA, USA). The analytical method was proven to be accurate and linear in the range of 10–250 ng/mL.

### 2.5. Adsorption Efficiency of Biochars on AFB1 in Buffer Solutions

#### 2.5.1. AFB1 Stock Solution

AFB1 (purity ≥ 98%, Cayman Chemical Company, Ann Arbor, MI, USA) stock solution was prepared by dissolving all the powder from one vial (1 mg) in DMSO and adjusting the final volume to 20 mL to obtain a concentration of 49,000 ng/mL (ppb). This stock solution was subsequently used to perform adsorption studies.

#### 2.5.2. In Vitro Adsorption Studies

The adsorption capacity of B-OP and B-GL was evaluated at a concentration of 0.05% (*w*/*v*) using AFB1 solutions prepared in acetate (pH 5.0), chloride (pH 1.2), and phosphate (pH 6.8) buffers, as they correspond to the three main compartments of the avian gastrointestinal tract: the crop, proventriculus, and an intestinal section, respectively. The AFB1 solutions were prepared by taking 25 μL of the stock solution and bringing it to a volume of 50 mL with buffer solutions to achieve a concentration of 250 ng/mL (ppb) and a DMSO concentration of 0.05%. Briefly, 2.5 mg of B-OP or B-GL was placed in 15 mL polypropylene Falcon tubes, followed by the addition of 5 mL of the buffer solutions containing AFB1. The tubes were kept in agitation at 19 rpm using an orbital shaker (model 3500, VWR International, Radnor, PA, USA) for 15 min at 40 °C in a biochemical oxygen demand incubator (model 2020, VWR, Houston, TX, USA) to reach equilibrium, in accordance with previous studies [28,29]. The samples were subsequently centrifuged at 3500 rpm for 10 min at 4 °C (Microfuge R20, Beckman Coulter Life Sciences, Palo Alto, CA, USA), and then the supernatants were passed through 0.2 μm polytetrafluoroethylene filters (PTFE Acrodysc; Gelman Sciences, Ann Arbor, MI, USA) for further analysis by UPLC. The studies were performed in sextuplicate; the buffer solution with AFB1 was considered a positive control, and the buffer solution without AFB1 but with B-OP or B-GL was considered a negative control.

#### 2.5.3. Mechanistic Insights into AFB1 Adsorption by Biochars Using FTIR Analysis

Following the in vitro adsorption studies of AFB1 in buffer solutions, a complementary analysis was performed to elucidate the possible mechanism of AFB1 adsorption by BCS. Briefly, the supernatant from the tubes centrifuged in step 2.6.1 was carefully decanted until only the BCS remained at the bottom of the tubes. The tubes were then placed in an oven and maintained at 60 °C for 48 h to ensure complete drying of the BCS. After the drying process was completed, the BCSs with and without AFB1 were analyzed by FTIR under the same conditions described in Section 2.3.3, and the obtained spectra were compared to determine the adsorption mechanism by considering the intensity, shape, or displacement of the characteristic bands of the functional groups.

### 2.6. Adsorption Studies of AFB1 Using an In Vitro Avian Model

#### 2.6.1. Optimization of the In Vitro Model

##### Effect of the Amount of Corn on the Adsorption of AFB1

The adsorption capacity of B-OP and B-GL on AFB1 was evaluated in an in vitro model that simulates the physiological conditions of the avian gastrointestinal tract in terms of pH, enzymatic activity, the presence of feed, and retention time, as described in previous publications, with slight modifications [32,33]. However, before conducting the AFB1 adsorption studies, the effect of the amount of corn (feed) in the in vitro model was evaluated in order not to compromise the results. In this regard, to evaluate the effect of the feed matrix, different amounts of corn (25, 250, 500, 1000, and 2000 mg) were placed in 50 mL Falcon tubes in the absence of biochars (B-OP or B-GL) and digestive enzymes (pepsin–pancreatin). Subsequently, 46 µL of an AFB1 solution at a concentration of 49,000 ppb was added to each tube, reaching a concentration of 250 ppb at the end of the model (intestinal section). Each compartment simulation of the model and the sample treatment were performed under the conditions described in Section 2.6.2.

##### Effect of Digestive Enzymes on AFB1 Adsorption

These studies were also conducted using the in vitro avian model. The same temperature and incubation time conditions were maintained, as well as the addition of media to achieve the different pH conditions. However, corn was not included. Briefly, in a 50 mL Falcon tube, 46 µL of an AFB1 solution at a concentration of 49,000 ppb was added to achieve a concentration of 250 ppb at the end of the model (intestinal section). Subsequently, the appropriate media were added to achieve the different pH conditions with or without the inclusion of digestive enzymes (pepsin–pancreatin) and B-OP or B-GL at 0.05%. In the case of the compartment that simulates the proventriculus, 3000 U/g of pepsin corn was added, and, for the intestinal section, 8× pancreatin was added at a concentration of 6.84 mg/g of corn. The amount of enzymes added was based on the inclusion of 125 mg of corn in the in vitro model since it was the one that showed the lowest adsorption of AFB1. Once the model was completed, the sample treatment was carried out under the conditions described in Section 2.6.2.

#### 2.6.2. Adsorption Studies in the In Vitro Avian Model

In these studies, two reference materials were included: a non-commercial zeolite (size < 250 μm) and a commercial *Saccharomyces cerevisiae* yeast cell wall (SafMannan, Lesaffre Iberica S.A., Valladolid, Spain). The non-commercial zeolitic material used in this study was a natural zeolitic mineral obtained from Guerrero, Mexico, which was further characterized in our previous study [30]. The material showed a predominantly heulandite-type mineralogical composition, with clinoptilolite, quartz, calcite, and magnetite as additional crystalline phases. A biochemical oxygen demand incubator (model 2020, VWR, Houston, TX, USA) was used for these studies, which was set to a temperature of 40 °C and equipped with an orbital shaker operating at 19 rpm (VWR, Houston, TX, USA). The 50 mL polypropylene Falcon tubes used in the model, containing corn (as feed), enzyme solutions, AFB1, and B-OP or B-GL, were held at a 30° angle of inclination to facilitate homogenization.

To simulate the crop, the first compartment of the simulated gastrointestinal tract, 125 mg of ground corn that was previously sieved through a No. 60 mesh to homogenize the particle size, 4.5 mg of B-OP or B-GL (0.05%), and 46 µL of an AFB1 solution at a concentration of 49 ppm (µg/mL) were placed in a 50 mL Falcon tube to reach a concentration of 250 ng/mL at the end of the model (intestinal section). Subsequently, 5 mL of 0.002 M HCl was added, resulting in a pH between 5.0 and 5.2. Thereafter, the tubes were vigorously shaken using a vortex mixer and incubated for 30 min with continuous shaking. Once the first compartment was completed, 3000 U of pepsin was added for each g of feed contained in 1.25 mL of 0.130 M HCl to simulate proventriculus conditions and thus achieve a pH between 1.4 and 2.0. The tubes were shaken again and incubated for another 45 min under constant shaking. Finally, to simulate the intestinal section (pH 6.4–6.8), 6.84 mg of 8× pancreatin per g of feed was added to 3.25 mL of a 0.1 M sodium bicarbonate solution in each tube, and incubated and shaken for an additional 2 h.

After the model was completed (3 h 15 min), the tubes were centrifuged at 3500 rpm for 10 min at 4 °C (Microfuge R20, Beckman Coulter Life Sciences, Palo Alto, CA, USA), and an aliquot of the supernatant was collected and filtered through a 0.2 μm PTFE filter. The filtrate was placed in vials to determine the AFB1 concentration by UPLC. The determinations were performed in sextuplicate, including a positive control (without B-OP or B-GL), to calculate the percentage of AFB1 adsorbed under the simulated conditions. The percentage of AFB1 adsorbed was calculated using the following equation:

$$Adsorption\left(\%\right)=\frac{\left(C_{i}-C_{s}\right)}{C_{i}}\times 100$$

where *C_i_* is the concentration of AFB1 in the positive control samples (ng/mL), and *C_s_* is the concentration of AFB1 in the samples with adsorbents (ng/mL).

### 2.7. Statistical Analysis

Data derived from the AFB1 adsorption studies in buffer solutions, as well as from the in vitro avian model and its optimization (effect of the amount of corn on the adsorption of AFB1 and effect of digestive enzymes on the adsorption of AFB1), were evaluated by a one-way analysis of variance (ANOVA), followed by a Tukey post hoc test (*p* < 0.05) using JMP^®^ Student Edition, version 19.0.3 (JMP Statistical Discovery LLC, Cary, NC, USA), and GraphPad Prism version 10.4.2 (GraphPad Software, San Diego, CA, USA). Before performing the ANOVA, compliance with the assumptions of normality and homoscedasticity was verified using the Shapiro–Wilk and Levene tests, respectively, at a 95% confidence level. Data are presented as mean ± standard error (SE).

## 3. Results

### 3.1. Characterization of Biochar

#### 3.1.1. Particle Size Analysis

The results of the laser diffraction particle size analysis of the biochars derived from orange peel (B-OP) and guava leaves (B-GL) are shown in Table 1. The average particle size of B-OP was smaller (55.30 µm) than that obtained for B-GL (82.88 µm). Furthermore, the variation in size was also less in the case of B-OP, reflecting a slightly more homogeneous distribution than B-GL. The yields of these carbonaceous materials were 7.52% and 4.93% for B-OP and B-GL, respectively.

#### 3.1.2. Surface Morphology of Biochars

**Figure 1:**
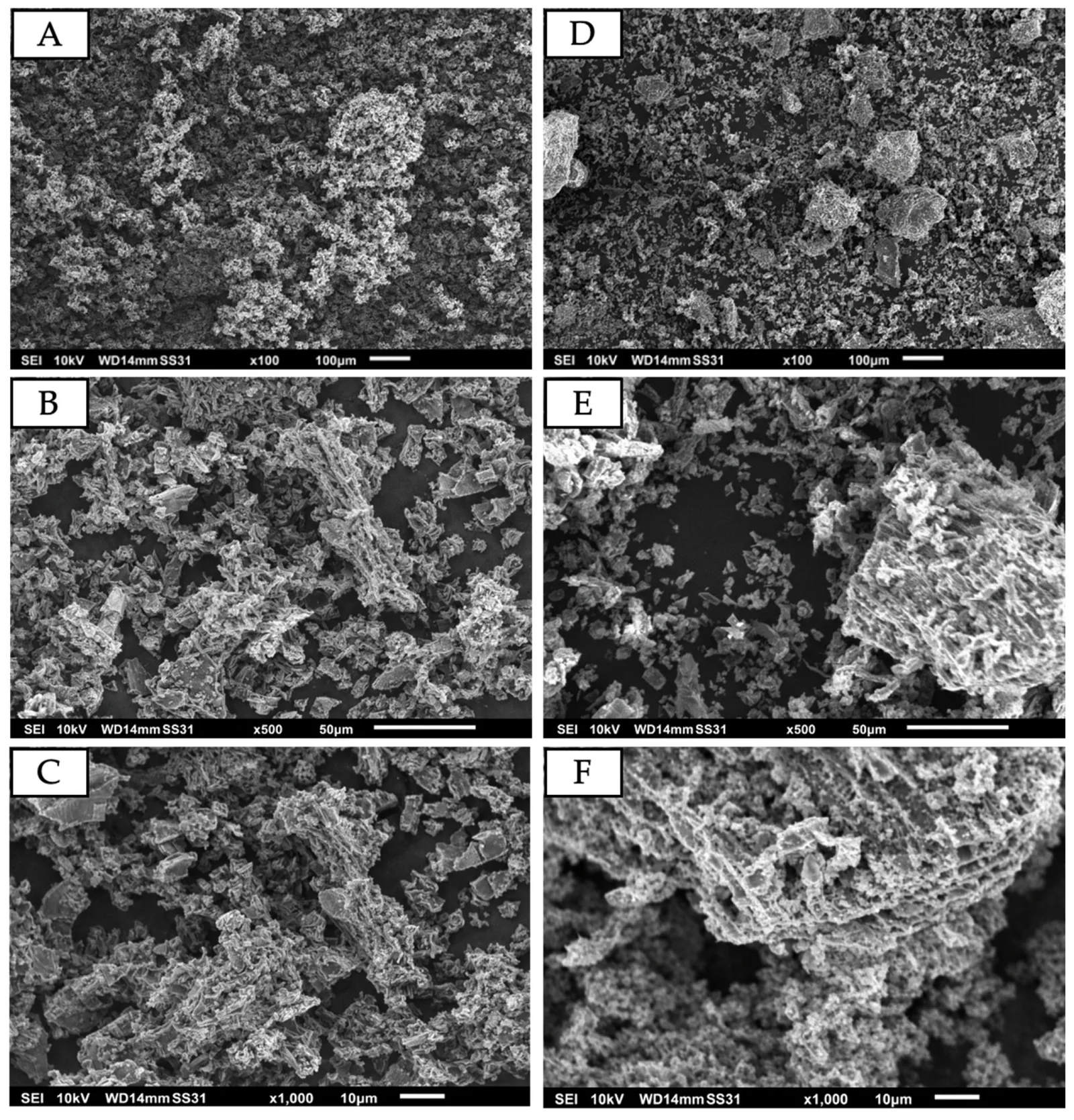
Scanning electron micrographs (SEMs) of biochars derived from agro-industrial waste of orange peel (B-OP) obtained at 100× (**A**), 500× (**B**), and 1000× (**C**) and guava leaves (B-GL) at 100× (**D**), 500× (**E**), and 1000× (**F**).

The surface morphology and microstructural organization of B-OP and B-GL were evaluated by scanning electron microscopy (SEM) at magnifications of 100×, 500×, and 1000× (Figure 1). At magnifications of 100×, 500×, and 1000× (Figure 1A–C), fragments with flat faces and fractured regions are distinguished, but a more defined and heterogeneously distributed micrometric porous network is observed, which is associated with the pyrolysis process of hemicellulose, cellulose, and lignin during the biochar production process. Meanwhile, the micrographs corresponding to B-GL at magnifications of 100× and 500× (Figure 1D,E) show aggregates of particles of different sizes with laminar and fibrillar regions, as well as mesh-like structures. At a higher magnification (1000×), a rough surface and a highly porous irregular structure on the order of micrometers can be observed, with the presence of interconnected cavities (Figure 1F), which is favorable for making the adsorption processes more efficient.

#### 3.1.3. Fourier Transform Infrared Spectroscopy with Attenuated Total Reflection (FTIR-ATR) Analysis

**Figure 2:**
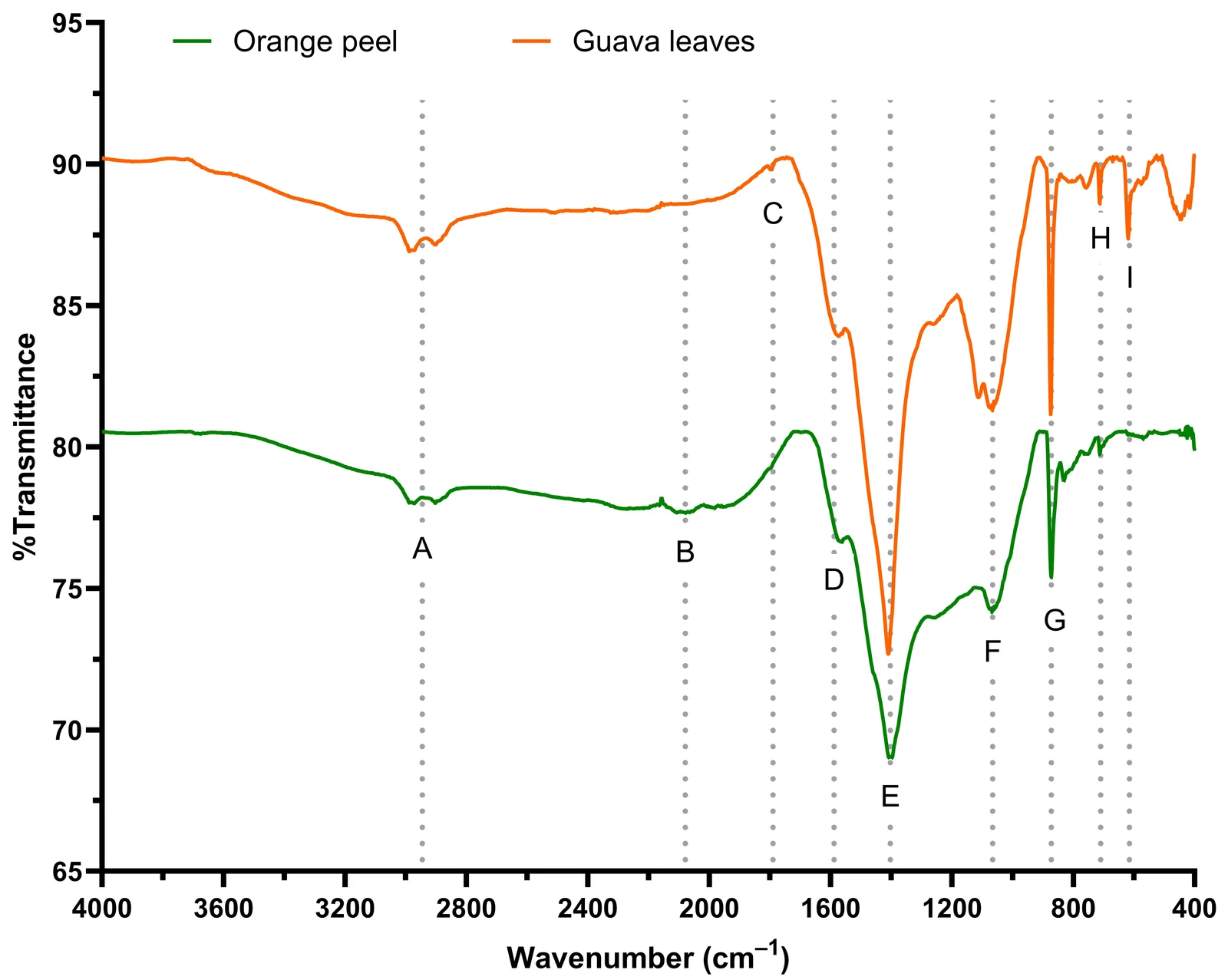
Fourier transform infrared (FTIR) spectra of biochars derived from agro-industrial waste of orange peel (B-OP) and guava leaves (B-GL).

The FTIR-ATR spectra of B-OP and B-GL obtained in the spectral range of 4000 cm^−1^ to 400 cm^−1^ show nine important zones in which the bands corresponding to the functional groups present in these biochars are found (Figure 2 and Table 2). The bands located between 2985 cm^−1^ and 2900 cm^−1^ correspond to the aliphatic C–H stretching vibrations of the aldehyde groups present in both biochars (B-OP and B-GL). At 2085 cm^−1^, a low-intensity band associated with the stretching of nitrile groups (–C≡N) is observed, but only in B-OP. Conversely, only in B-GL is there a band at 1800 cm^−1^, which is associated with aromatic C–H stretching vibrations. In both biochars, the band located at 1580 cm^−1^ corresponds to aromatic and olefinic C=C stretching vibrations. Furthermore, the bands at 1405 cm^−1^ and 1060 cm^−1^ confirm the asymmetric COO− vibration and the C–O stretching of aryl ether bonds, respectively, in both B-OP and B-GL. Finally, in B-OP, a band is observed at 872 cm^−1^, which is associated with the C–H stretching vibrations of the phenyl ring, and, only in B-GL, the aromatic C–H stretching vibrations and C–C carbon skeleton are associated with the bands found at 713 cm^−1^ and 617 cm^−1^.

#### 3.1.4. Point of Zero Charge (pH_pzc_) and Isoelectric Point (PI)

**Figure 3:**
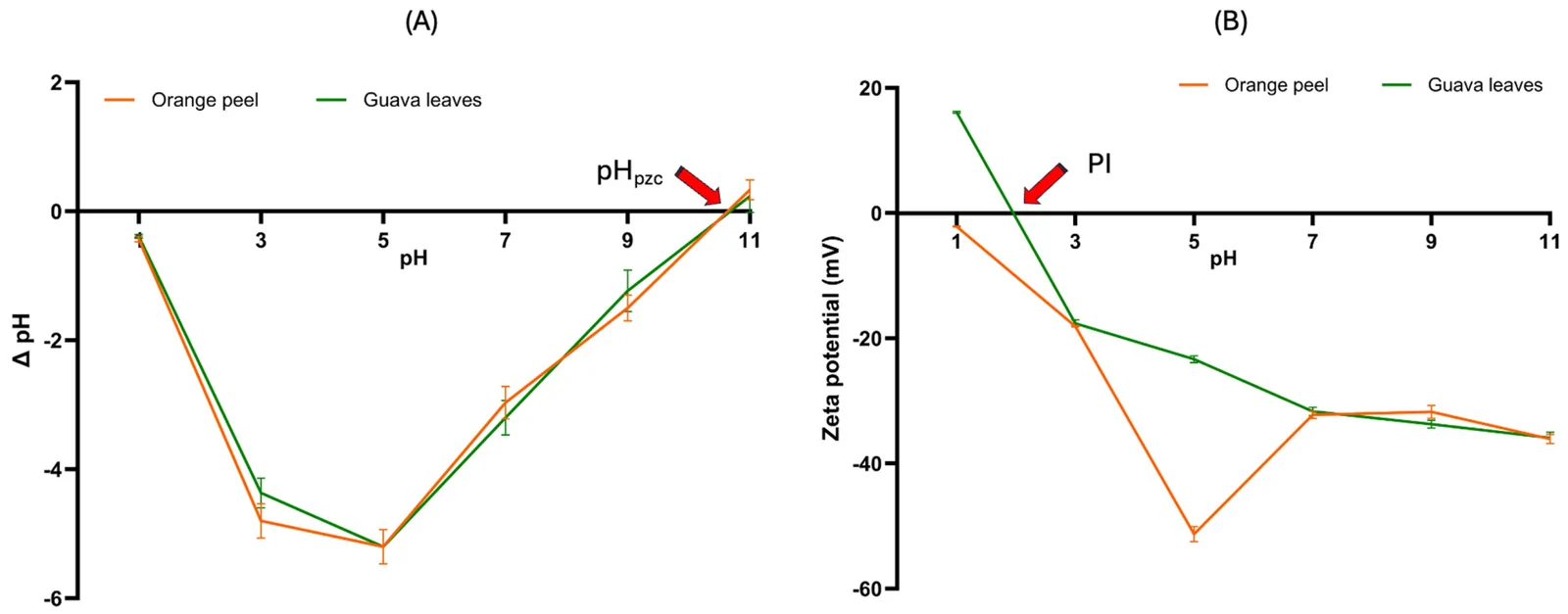
(**A**) Point of zero charge (pH_pzc_) and (**B**) zeta potential of biochars derived from agro-industrial waste of orange peel (B-OP) and guava leaves (B-GL) at different pH values.

Complementing the FTIR analysis, the pH_pzc_ (Figure 3A) and PI (Figure 3B) of B-OP and B-GL were determined, since they are indicative of the magnitude of their surface charge due to the protonation and deprotonation of certain oxygenated surface groups. In the case of pH_pzc_, the two biochars exhibited similar behavior, reaching a value of 10.7 (Figure 3A), which means that, at this pH, the positive and negative charges are in equilibrium (net load of zero). Meanwhile, the zeta potential differed among the biochars. B-GL at pH = 1 showed a positive value (16.1 mV), and, as the pH increased, it became more negative (−35.9 mV at pH = 11). Furthermore, it had a PI of 1.9, meaning that, at this pH, the zeta potential was zero. In contrast, the zeta potential of B-OP was negative at all pH values evaluated, starting with a value of −2.1 mV at pH = 1 and reaching the most negative value at pH = 5 (−51.3 mV), but it did not present isoelectric points.

#### 3.1.5. Determination of Surface Area and Pore Size

The results of the surface area, pore volume, and pore size distribution of the biochars determined by the BET technique and the BJH method are shown in Table 3. B-OP showed the largest surface area (31.50 m^2^/g) but the smallest pore size (2.38 nm) when compared to B-GL, which showed a surface area and pore size of 9.74 m^2^/g and 6.24 nm, respectively. The surface area results correlate perfectly with the particle size results, since the smaller the size, the greater the surface area. Regarding pore volume, this was slightly higher in B-OP (0.038 cc/g) than in B-GL (0.033 cc/g).

### 3.2. Adsorption Efficiency of Biochars on AFB1 in Buffer Solutions

**Figure 4:**
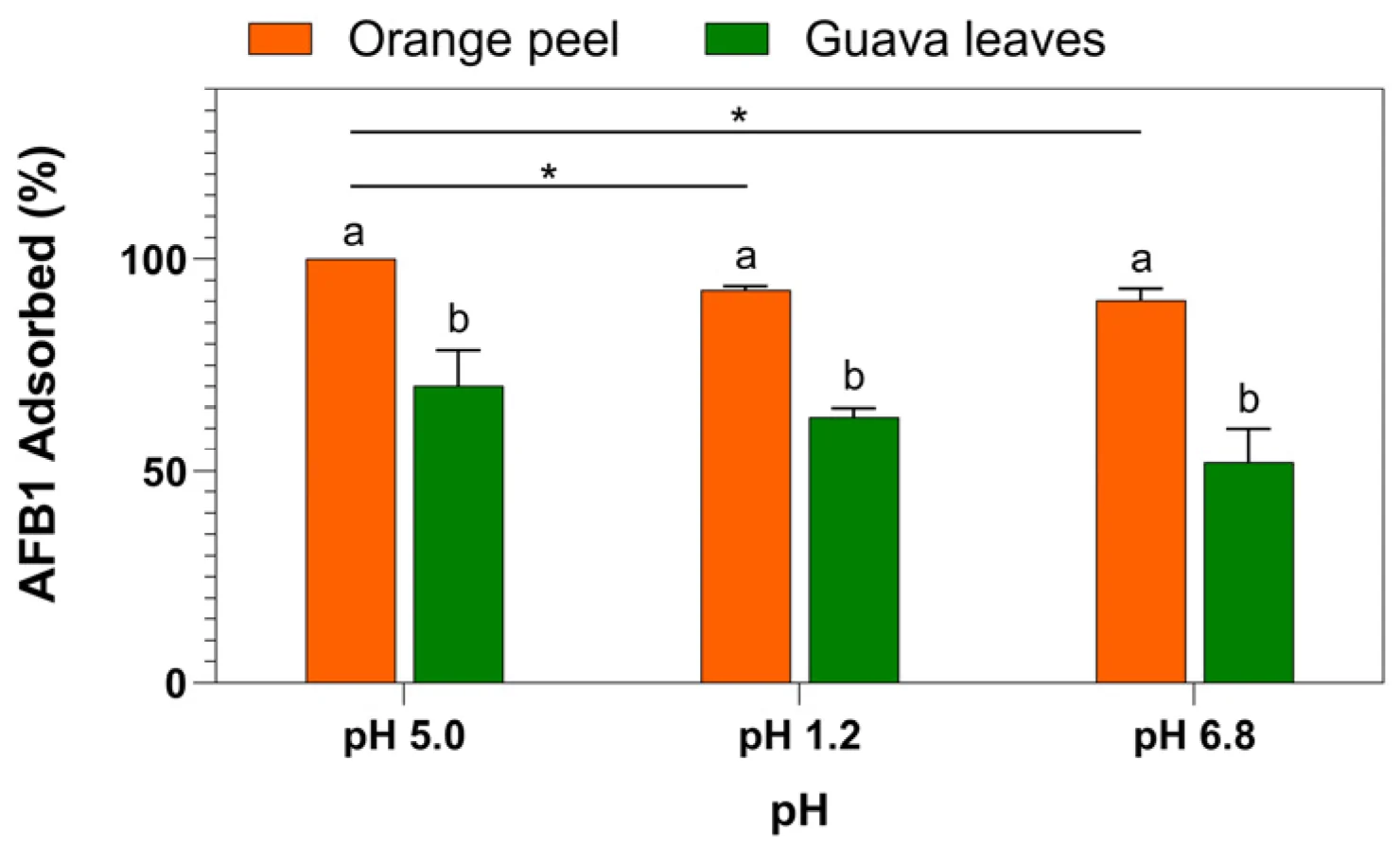
Percentage of aflatoxin B1 (AFB1) adsorbed by biochars derived from agro-industrial waste of orange peel (B-OP) and guava leaves (B-GL) at 0.05% at different pH levels, considering an AFB1 concentration of 250 ng/mL. ^a,b^ Bars with different letters are considered to show significant differences (*p* < 0.05). * Indicates significant differences between pHs considering B-OP (*p* < 0.05). Results are expressed as mean ± SE (*n* = 6).

The adsorption capacity of B-OP and B-GL on AFB1 in aqueous buffer solutions at pH 5.0, 1.2, and 6.8 is shown in Figure 4. The results showed that the adsorption capacity of B-OP was significantly higher than that of B-GL in the aqueous buffer solutions adjusted to pH 5.0, 1.2, and 6.8. Specifically, the adsorption efficiencies of B-OP at pH 5.0, 1.2, and 6.8 were 100.0%, 92.7%, and 90.3%, respectively, indicating a significant decrease in adsorption at pH 1.2 and 6.8 compared to at pH 5.0. In contrast, there were no significant differences in the adsorption efficiency of B-GL at pH 5.0 (70.0%), pH 1.2 (62.7%), or pH 6.8 (52.0%), but like B-OP, there was also a reduction in the adsorption of AFB1 with respect to pH. In this regard, for both biochars, the adsorption of AFB1 was greater at pH 5.

#### Mechanistic Insights into AFB1 Adsorption by Biochars

**Figure 5:**
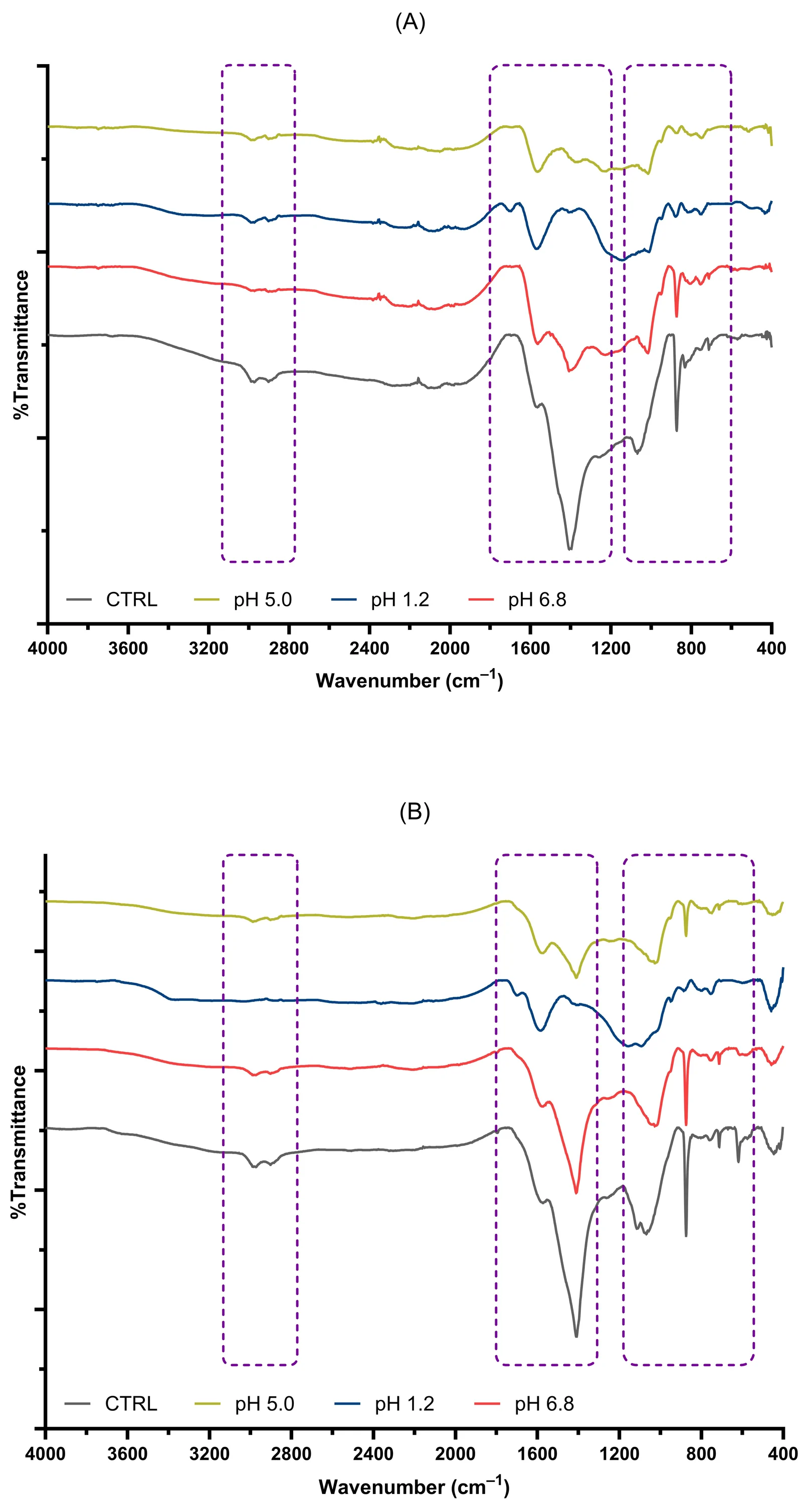
FTIR spectra of biochars derived from agro-industrial waste of orange peel (B-OP) (**A**) and guava leaves (B-GL) (**B**) before and after AFB1 adsorption studies in buffer solutions at pH 5.0, 1.2, and 6.8. The purple dotted lines indicate the regions where changes in spectral behavior occurred.

In order to elucidate the adsorption mechanisms of the biochars, FTIR spectra were obtained before and after evaluating their adsorption efficiency on AFB1 (Figure 5). In the FTIR spectra of B-OP, three regions were observed to show differences in their behavior before and after the AFB1 adsorption process (Figure 5A). In the first region, a reduction in the intensity of the band corresponding to the aliphatic C–H stretching vibration of the aldehyde groups (2985–2900 cm^−1^) was observed, but it cannot necessarily be considered one of the primary regions responsible for the adsorption processes. However, in the second region, more evident changes were observed, with decreases in intensity, widening, and displacement of the bands. In this region, the band corresponding to aromatic and olefinic C=C stretching vibrations (1580 cm^−1^) increased in intensity in B-OP exposed to AFB1 compared to that in the control B-OP, showing the highest intensity at pH 1.2, followed by pH 5.0 and 6.8. This behavior could be related to π–π stacking interactions between the aromatic rings of AFB1 and the aromatic C=C groups of B-OP. Furthermore, the band corresponding to the asymmetric vibration of COO− showed a greater reduction in intensity at pH 1.2, 5.0, and finally 6.8, suggesting that the greatest interaction between B-OP and AFB1 could be related to the formation of hydrogen bonds and dipole–dipole interactions. In the third region, the band corresponding to the C–O stretching of the aryl ether bonds (1060 cm^−1^) was reduced in intensity at pH 5.0 and 1.2, but at pH 6.8, no noticeable changes were observed compared to that in the B-OP control. Finally, the band of the C–H stretching vibration of the phenyl ring (872 cm^−1^) disappeared at pH 5.0 and 1.2, and at pH 6.8, it was reduced compared to that in the control B-OP. In both cases, these changes could be attributed to hydrophobic interactions.

Similarly to B-OP, the FTIR spectra of B-GL were modified (Figure 5B). In the region of 2985–2900 cm^−1^ (aliphatic C–H stretching vibration of aldehyde groups), reductions in the intensity of the bands were observed, being more evident at pH 5.0 and 1.2. Furthermore, the band at 1405 cm^−1^ (corresponding to the asymmetric vibration of COO−) decreased in intensity at pH 5.0 and 6.8, but a shift in the band occurred at pH 1.2. In contrast, the band attributed to aromatic C-H stretching vibrations (1800 cm^−1^) was modified, but only at pH 5.0. Regarding the C-O stretching vibration of the aryl ether bonds (1060 cm^−1^), only slight changes were observed at pH 5.0 and 6.8, while a notable broadening of the band occurred at pH 1.2. Finally, in the third region, reductions in band intensity were observed at 872 cm^−1^ (C–H stretching vibrations of the phenyl ring) and 713 cm^−1^ (aromatic C–H stretching vibrations) at pH 5.0 and 6.8, and they even disappeared at pH 1.2; additionally, the band at 617 cm^−1^ (carbonaceous C–C skeleton) disappeared at all three pHs.

### 3.3. Adsorption Efficiency of Biochars Toward AFB1 in an In Vitro Avian Model

#### 3.3.1. Optimization of the In Vitro Model

##### Effect of the Amount of Corn on the Adsorption of AFB1

**Figure 6:**
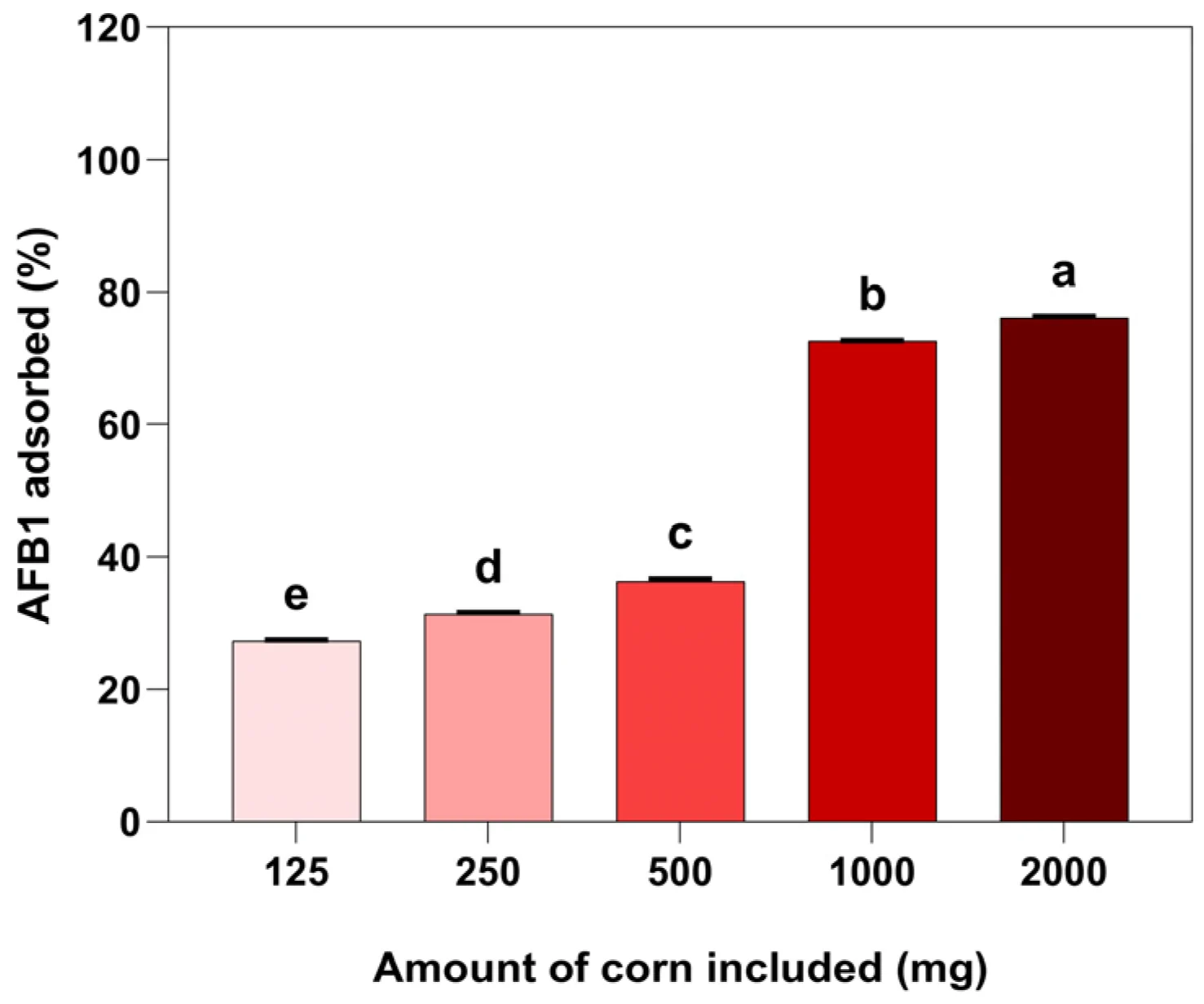
Percentage of AFB1 adsorbed as a function of the amount of corn included in the in vitro avian digestibility model. The initial concentration of AFB1 was 250 ng/mL. ^a–e^ Bars with different letters are considered to show significant differences (*p* < 0.05). Results are expressed as mean ± SE (*n* = 6).

The percentage of AFB1 adsorbed as a function of the amount of feed (corn) evaluated in the in vitro avian model is shown in Figure 6. At corn quantities of 125 mg and 250 mg, the adsorption of AFB1 was relatively low, with values between 27.3% and 31.3%, respectively. In particular, at 125 mg, a significantly lower adsorption was observed than in the rest of the treatments. Increasing the amount of corn to 500 mg resulted in significantly higher AFB1 adsorption (36.3%) than the amounts of 125 mg and 250 mg. Finally, at corn concentrations of 1000 mg and 2000 mg, a pronounced and significant increase in AFB1 adsorption was observed compared to the other concentrations, reaching values of 72.6% and 76.1%, respectively. However, the percentage of AFB1 adsorption was significantly higher when 2000 mg was used than when 1000 mg was used. The differences observed between all treatments confirm a direct dependence between the amount of corn and the adsorption of AFB1 in the in vitro avian model. Therefore, it was decided to use 125 mg of corn in the in vitro avian model since it was the amount that presented the lowest percentage of AFB1 adsorption.

##### Effect of Digestive Enzymes on AFB1 Adsorption

Table 4 shows the percentage of AFB1 adsorbed in the presence or absence of digestive enzymes (pepsin and pancreatin) considering the conditions of the in vitro avian model without corn. The results indicate that the addition of digestive enzymes, specifically pepsin in the compartment simulating the proventriculus and pancreatin in the intestinal phase, did not result in AFB1 adsorption, as no significant changes were observed in the percentage of AFB1 present in the medium compared to the medium without enzymes. However, when biochars were included, AFB1 adsorption was altered; that is, the presence of enzymes had a negative effect on adsorption by the biochars. In fact, AFB1 adsorption by B-GL was significantly more affected in the presence of digestive enzymes than that by B-OP.

#### 3.3.2. AFB1 Adsorption Efficacy in the Optimized In Vitro Model

**Figure 7:**
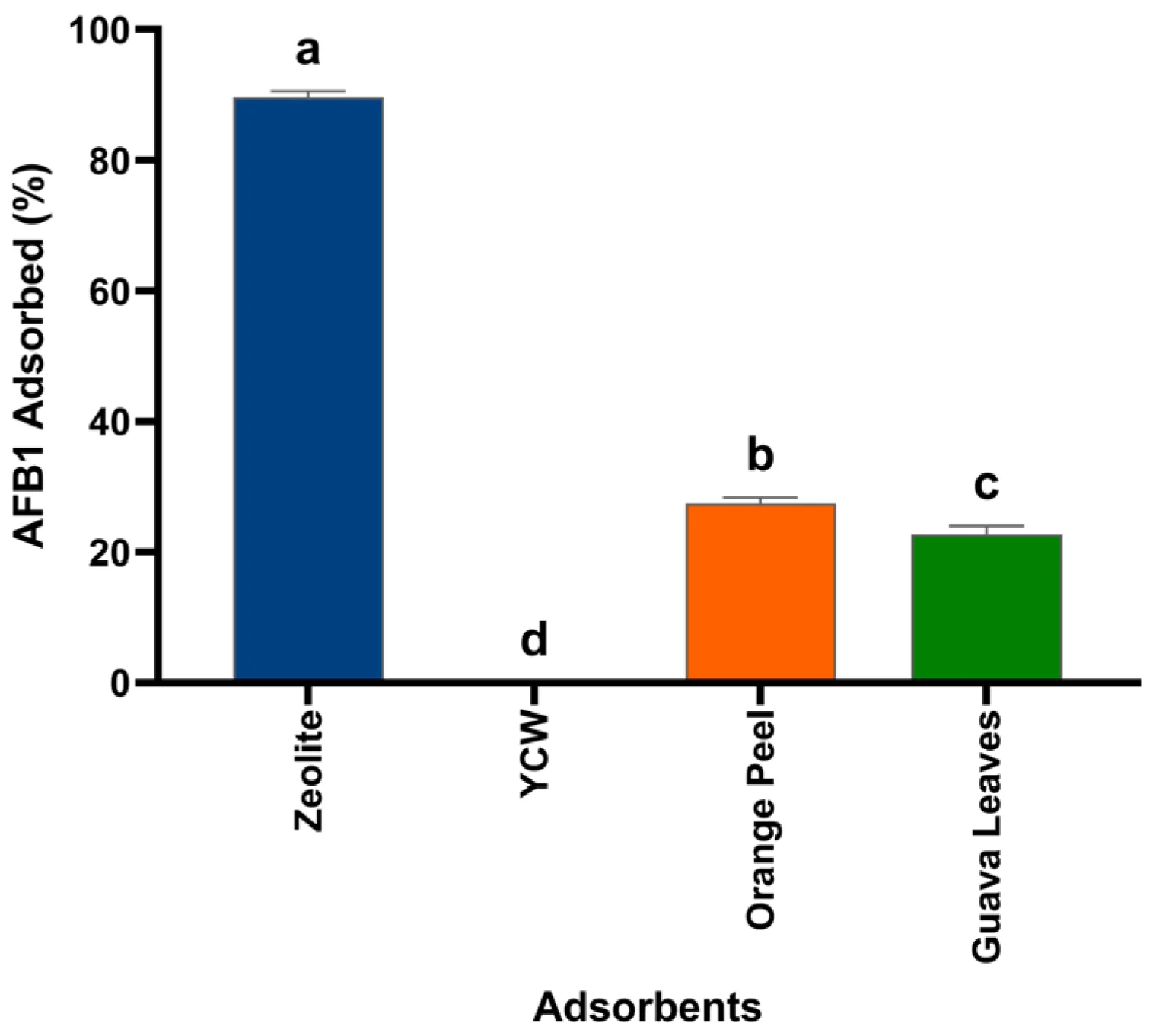
Percentage of aflatoxin B1 (AFB1) adsorbed by biochars derived from agro-industrial waste of orange peel (B-OP) and guava leaves (B-GL) at 0.05% in the in vitro avian digestion model, considering an AFB1 concentration of 250 ng/mL. ^a–d^ Bars with different letters are considered to show significant differences (*p* < 0.05). Results are expressed as mean ± SE (*n* = 6). YCW: yeast cell wall.

The results of the AFB1 adsorption capacity of B-OP and B-GL, as well as that of a commercial product based on yeast cell wall (YCW) and a non-commercial zeolite, in the optimized in vitro avian model are shown in Figure 7. The adsorption results in the in vitro avian model correspond only to the end of the model compartment (intestinal section). Among the different treatments evaluated for AFB1 removal, YCW showed no detectable adsorption capacity. In contrast, the non-commercial zeolite exhibited the best AFB1 adsorption capacity, achieving 89.6% removal. In the case of the biochars, they showed intermediate adsorption, but B-OP was statistically more efficient since it removed 27.4% of AFB1, whereas B-GL removed 22.7% of AFB1. These results differ considerably from those obtained in buffer solutions because the in vitro model considers the effect of feeding, pH conditions, enzyme activity, and residence times in the avian gastrointestinal tract. In fact, the AFB1 adsorption capacity of B-OP was reduced by 62.9%, while that of B-GL was reduced by 29.5%.

## 4. Discussion

Agro-industrial waste represents one of the main environmental problems associated with agri-food production systems due to the large volumes generated annually and its limited valorization [34]. In particular, orange peels and guava leaves are two agro-industrial waste products widely generated by the citrus industry [24,25] and the increasing availability of guava leaf biomass due to the expansion of guava production [27], respectively. The interest in these materials lies in their potential value as adsorbents, contributing to the reduction of their environmental impact and taking advantage of their structural and chemical properties for the removal of contaminants [35]. In this sense, their high contents of cellulose, hemicellulose, and lignin make them attractive precursors for obtaining biochars [36]. Considering this background, the use of biochars added to feed for the removal of AFB1 is relevant to ensure food security. In fact, the global presence of AFB1 in feed intended for production animals has been reported to be between 5.9% and 82.2%, and in the poultry industry alone, 20% of feed is contaminated with this mycotoxin [37,38]. Although the incidence of AFB1 in poultry farms is not very high and only around 5% had values higher than those allowed (2 µg/kg), it is known that the adsorption of AFB1 is high (>80%) in the small intestine of poultry, which negatively impacts production parameters, causing economic losses [9]. Furthermore, a greater concern is the increased biotransfer of this mycotoxin from feed to animal tissues and even to by-products such as eggs, which can have effects on humans [38]. Therefore, the present study aimed to obtain two sustainable and low-cost biochars from agro-industrial waste of orange peel (B-OP) and guava leaves (B-GL) to evaluate their efficiency in the removal of AFB1 in an in vitro avian model.

In the present study, the two biochars obtained were characterized physicochemically. B-OP showed a smaller average particle size (55.30 ± 32.20 µm), as well as a larger surface area (31.50 m^2^/g), than B-GL (82.88 ± 43.80 µm and 31.50 m^2^/g, respectively) (Table 1 and Table 3). The differences in particle size were also supported by SEM analysis since smaller heterogeneous porous structures were observed in B-OP than in B-GL (Figure 1). Although it is known that the average particle size of biochar decreases as the pyrolysis temperature increases due to the weakening of the macromolecular structure and the formation of fragile particles prone to breakage [39], the conditions for obtaining both biochars were the same. In this sense, differences in the composition of lignocellulosic materials in the biomasses used for the production of biochars were responsible for the differences in particle size [40]. In fact, it has been reported that cellulose promotes the formation of micropores through graphitization, while the intermediates produced by the decomposition of hemicellulose and lignin result in a poor pore structure and a reduced actual specific surface area [41,42]. In this context, the results obtained for pore size in B-OP (2.38 nm) and B-GL (6.24 nm) are supported since it has been reported that orange peels contain a higher percentage of cellulose (69.1%), followed by lignin (19.8%) and hemicellulose (9.0%) [43]. Meanwhile, guava leaves can contain 5.5–35.4%, 17.8–35.3%, and 11.0–34.1% cellulose, lignin, and hemicellulose [44], respectively. These characteristics of size, surface area, and pore size are relevant to food safety applications of biochars since they can be used for the removal of AFB1 in poultry feed.

The biochars were also characterized by FTIR to identify the functional groups that could be involved in the adsorption processes (Figure 2 and Table 2). The FTIR spectra showed differences between the biochars, but both retained functional groups associated with lignocellulosic precursors, specifically oxygenated groups and aromatic structures, which could actively participate in the AFB1 adsorption process [45]. Furthermore, the evaluation of the pH_pzc_ and PI showed differences in the magnitude of their surface charge due to the protonation and/or deprotonation of certain oxygenated surface groups [46]. Although there were no differences between the pH_pzc_ (10.7) of the biochars (Figure 3A), the PI differed (Figure 3B), since B-OP presented a value of 1.9, while B-GL did not present a PI. A difference between the pH_pzc_ and PI has been reported in porous carbonaceous materials, and, usually, the PI is lower than the pH_pzc_ mainly due to the preferential oxidation of external surface sites when exposed to air at room temperature [46]. This is because the PI only reflects surface charges, not those inside the pores; that is, it can be useful for determining the stability of materials in a solution [46]. Meanwhile, the pH_pzc_ reflects the net surface charge, including both external and internal charges [46]. Therefore, evaluation and comparison of the pH_pzc_ and PI are useful for assessing the relative influence of external or internal loads during adsorption processes.

Like the differences observed in the physicochemical characterization of the biochars, in vitro adsorption studies performed in buffer solutions at pH 5.0, 1.2, and 6.8 showed different AFB1 removal efficiencies between B-OP and B-GL, as well as at the three pHs evaluated (Figure 4). B-OP showed the highest AFB1 removal efficiency, achieving 100.0% adsorption at pH 5.0, 92.7% at pH 1.2, and 90.3% at pH 6.8. In contrast, B-GL exhibited a lower AFB1 removal efficiency than B-OP, and it was pH-dependent, achieving 70.0%, 62.7%, and 52.0% at pH 5.0, 1.2, and 6.8, respectively. However, the greatest removal of AFB1 was observed at pH 5.0 for both biochars, which may be supported by the pH_pzc_ since, at pH 5, the greatest number of negative charges was found, which suggests that there were greater interactions between the surface functional groups of the biochars and AFB1. Furthermore, the changes observed in the FTIR spectra after the AFB1 removal studies support the involvement of multiple interactions (Figure 5; hydrophobic interactions, van der Waals forces, hydrogen bonds, dipole–dipole intermolecular interactions, π–π stacking interactions, and, in certain cases, electrostatic interactions) [47,48,49], but it is a fact that the main mechanism of AFB1 removal is associated with the pore size of the biochars. In this regard, mesoporous materials with diameters between 2 nm and 50 nm have been reported to exhibit better AFB1 adsorption due to a better fit of the AFB1 molecule, whose width ranges from 1.08 nm to 1.28 nm [50]. Therefore, the best fit of AFB1 likely occurred in the pore of B-OP (2.38 nm) since it is smaller than that of B-GL (6.24 nm).

Although in vitro models that simulate pH conditions provide useful information on the ability to remove contaminants such as AFB1 using adsorbent materials, they do not consider the dynamic conditions of the gastrointestinal tract such as the presence of feed, digestive enzymes, and residence time in each compartment. Consequently, the contaminant removal efficiency determined in simple systems is not able to accurately reflect the performance of adsorbents under more complex gastrointestinal conditions (in animals). Therefore, an in vitro avian model was optimized, which simulated the conditions of the gastrointestinal tract of broilers in terms of feed presence, pH, enzyme activity, and residence time in the crop (pH 5.0), proventriculus (pH 1.2 with pepsin), and an intestinal section (pH 6.8 with pancreatin). The first part of the model optimization process consisted of selecting the amount of corn, the main ingredient in the diets of the broilers, since it has been reported that its composition contains cellulose, xylan, lignin, and arabinoxylan, which can adsorb AFB1 and lead to overestimation of the results [51]. The results obtained showed that the lowest adsorption of AFB1 (27.3%) was obtained with 125 mg of corn in the model (Figure 6), so this amount was selected to maximize the capacity to discriminate AFB1 adsorption among the biochars. In the second part of the model optimization process, the effect of digestive enzymes, pepsin and pancreatin, on AFB1 adsorption was evaluated, both with and without the presence of B-OP or B-GL. To the best of our knowledge, this is the first study to report that digestive enzymes do not have the capacity to adsorb AFB1, as shown in Table 4. Only one study reported that pepsin has the ability to reduce the adsorption efficiency of AFB1 from montmorillonite, a clay material, but its adsorption capacity improved with the inclusion of nutrients (vitamin B1), which were intercalated in the adsorbent material [52]. However, since digestive enzymes are proteins, they can interact with biochar surfaces, competing for active adsorption sites, blocking micropores, or altering the way by which AFB1 binds to the biochar, potentially resulting in reduced adsorption efficiency, as observed in our assays (Table 4). The optimized model was used to evaluate the efficiency of AFB1 removal from B-OP and B-GL, and two controls were included. The results of AFB1 removal in the avian in vitro model differed considerably from those of the model that only considered buffer solutions. However, the AFB1 removal efficiency of B-OP was significantly higher (27.4%) than that of B-GL (22.7%) when considering only the intestinal section, which is the most important (Figure 7). The reduction in the AFB1 removal capacity of B-OP and B-GL could be explained by the occupation or physical obstruction of the active sites of the biochars, as well as the blocking of micropores by the corn components and digestive enzymes (pepsin and pancreatin) used in the model (non-digestible fiber, lignin, and cellulose), making them less effective [53]. Furthermore, it has been reported that the constituent components of corn (cellulose, xylan, lignin, and arabinoxylan) are capable of adsorbing AFB1, suggesting a potential competitive process with the biochars [51]. Although the AFB1 adsorption efficiency of the biochars was reduced in the avian in vitro model, they were able to remove 68.5 ppb (B-OP) and 56.8 ppb (B-GL). These values can be considered biologically significant, as they exceeded the 20 ppb limit for AFB1 in feed materials and complementary feeds established by the Codex Alimentarius Commission’s guidelines [54,55]. Thus, these biochars could remove AFB1 at levels up to approximately three times the permissible limits in feed, which can be influenced by various natural conditions, such as the protein and fat contents and composition of the food [55]. In the case of zeolite, the removal of AFB1 was more effective, but it is also known that it is more nonspecific, so it can compromise the growth of broiler chickens due to mechanisms such as delay in intestinal transit, immobilization of enzymes, and alterations in the intestinal microbiota [56,57]. Unlike zeolite and biochars, YWC exhibited very low AFB1 removal, which has been described as pH-dependent, being more effective at acidic pH. Furthermore, it has fewer binding sites, which can be more easily blocked by corn components [58]. Therefore, biochars, especially B-OP, could be positioned as safer and more sustainable alternatives with a potentially low risk of nutrient binding [19,59]. However, this potential advantage requires future experimental confirmation and compositional analysis, given that biochars are known to contain potentially toxic contaminants, such as heavy metals, polycyclic aromatic hydrocarbons, and volatile and/or easily leachable compounds [60].

### Limitations and Future Directions

While this study presents interesting results regarding AFB1 adsorption using biochars, it also reveals certain limitations that should be considered when planning future studies. First, adsorption isotherms for the biochars were not obtained, as AFB1 adsorption was performed using only a single biochar concentration (0.05%). Secondly, AFB1 adsorption by the biochars in the in vitro avian model was evaluated only in the intestinal section (pH 6.8); therefore, to better understand AFB1 adsorption, it would also need to be evaluated at pH 5.0 (crop) and 1.2 (proventriculus). Finally, the results obtained in the present study must be validated in animal models to determine the efficiency of the biochars in adsorbing AFB1 from contaminated feed.

## 5. Conclusions

The utilization and valorization of agro-industrial waste such as orange peel and guava leaf derivatives for the synthesis of biochars (B-OP and B-GL, respectively) represent a significant advance in the application of the circular economy to food security, since the conversion of these wastes into functional adsorbents not only reduces the environmental impact associated with their massive accumulation but also offers a viable and low-cost alternative for the removal of contaminants such as AFB1 in animal feed. Although adsorption studies in buffer solutions demonstrated AFB1 removal capacities of up to 100%, in the in vitro avian model, it was shown that the presence of a feed matrix (corn) generated competition for the active sites and micropores of the biochars, reducing their efficiency. However, despite this interference, B-OP proved to be statistically superior in the removal of AFB1 (68.5 ppb) compared to B-GL (56.8 ppb), mainly due to combined mechanisms such as a smaller pore size and larger surface area, demonstrating biological relevance given that food products must not exceed the 20 ppb limit. Further studies are required to evaluate their safety, effects on nutrient availability, and in vivo efficacy before considering their application as feed additives. Overall, these findings demonstrate the potential of biochars, particularly B-OP, as sustainable adsorbents for AFB1 under in vitro conditions and lay the groundwork for future research aimed at determining their applicability as a strategy to mitigate AFB1-related issues in poultry production systems.

## Figures and Tables

**Table 1:** Particle size of biochars derived from agro-industrial waste of orange peel (B-OP) and guava leaves (B-GL) determined by laser diffraction (DLS).

Biochar	Mean ± SD (µm)	Median (µm)	D_10_ (µm)	D_90_ (µm)
B-OP	55.30 ± 32.20	50.42	16.14	102.10
B-GL	82.88 ± 43.80	84.93	21.28	140.50

SD: standard deviation.

**Table 2:** Functional groups associated with the characteristic bands of biochars derived from agro-industrial waste of orange peel (B-OP) and guava leaves (B-GL).

Band	Wavenumber (cm^−1^)	Functional Group
A	2985–2900	Aliphatic C–H stretching vibration of aldehyde groups.
B	2085	Stretching of nitrile groups (–C≡N).
C	1800	Aromatic C–H stretching vibrations.
D	1580	Aromatic and olefinic C=C stretching vibrations.
E	1405	Asymmetric vibration of COO−.
F	1060	C–O stretching of aryl ether bonds.
G	872	C–H stretching vibrations of the phenyl ring.
H and I	713 and 617	Aromatic C–H stretching vibrations and carbonaceous C–C skeleton.

**Table 3:** Surface area and pore size of biochars derived from agro-industrial waste of orange peels (B-OP) and guava leaves (B-GL).

Biochar	Surface Area (m^2^/g)	Pore Volume (cc/g)	Pore Size (nm)
B-OP	31.50	0.038	2.38
B-GL	9.74	0.033	6.24

**Table 4:** Residual aflatoxin B1 (AFB1) after adsorption studies in the in vitro avian digestibility model without corn as feed, and in the presence or absence of digestive enzymes and biochars at 0.05%. The initial concentration of AFB1 was 250 ng/mL. Results are expressed as mean ± SE (*n* = 6).

Treatment	Residual AFB1 (%)
Media + AFB1 + NDE	101.60 ± 2.88 ^a^
Media + AFB1 + DE	98.94 ± 1.47 ^a^
Media + AFB1 + NDE + B-OP	0.00 ± 0.00 ^e^
Media + AFB1 + DE + B-OP	12.76 ± 0.35 ^d^
Media + AFB1 + NDE + B-GL	22.40 ± 1.87 ^c^
Media + AFB1 + DE + B-GL	42.45 ± 0.62 ^b^

NDE: no digestive enzymes; DE: digestive enzymes; B-OP: biochars derived from agro-industrial waste of orange peel; B-GL: biochars derived from agro-industrial waste of guava leaves (B-GL). ^a–e^ Values within rows with different superscripts show significant differences (*p* < 0.05). Results are expressed as mean ± SE (*n* = 6).

## Data Availability

The original contributions presented in the study are included in the article; further inquiries can be directed to the corresponding authors.

## Abbreviations

The following abbreviations are used in this manuscript:

AFB1	Aflatoxin B1
BET	Brunauer–Emmett–Teller
BJH	Barrett–Joyner–Halenda
B-OP	Biochar from agro-industrial waste of orange peel
B-GL	Biochar from agro-industrial waste of guava leaves
FTIR-ATR	Fourier Transform Infrared Spectroscopy with Attenuated Total Reflection
pH_pzc_	Point of zero charge
PI	Isoelectric point
PTFE	Polytetrafluoroethylene
SEM	Scanning electron microscopy
UPLC	Ultra-Performance Liquid Chromatography
